# Informing Pandemic Preparedness Through a Digital Global Health Security Library

**DOI:** 10.1089/hs.2021.0141

**Published:** 2022-06-17

**Authors:** Ellen P. Carlin, Ellie Graeden, Hailey Robertson, Rebecca Katz

**Affiliations:** Ellen P. Carlin, DVM, is an Assistant Research Professor, Center for Global Health Science and Security, Georgetown University, Washington, DC.; Ellie Graeden, PhD, is an Associate Research Professor (Adjunct), Center for Global Health Science and Security, Georgetown University, Washington, DC.; Rebecca Katz, PhD, MPH, is a Professor, Center for Global Health Science and Security, Georgetown University, Washington, DC.; Ellie Graeden is also Chief Operating Officer, Talus Analytics, Boulder, CO.; Hailey Robertson is a Researcher and Data Analyst, Talus Analytics, Boulder, CO.

**Keywords:** COVID-19, Epidemic management/response, Infectious diseases, National strategy/policy, Public health preparedness/response

COVID-19 has caused a moment of reckoning for the international community. For decades, experts in national security, public health, and global health security have warned of pandemic risk and recommended strategies to prevent and mitigate infectious disease outbreaks. Working across governments, international organizations, and the private and nonprofit sectors, these experts issued threat assessments, assessed capability gaps, developed pandemic preparedness plans, and created global frameworks to mitigate and manage biological risk. With over 6 million lives lost to COVID-19 as of March 17, 2022,^1^ there is a need to assess how governments and organizations translated knowledge into preparedness efforts.

Many studies have analyzed governments' responses to COVID-19 and previous outbreaks,^2-4^ but few have attempted to link these efforts to the warnings and recommendations of pandemic experts.^5^ Identification and analysis of myriad publications that reflect prepandemic activity may offer clues to both best practices and missed opportunities for infectious disease preparedness. However, such deconstruction of how the pandemic unfolded in the context of the actions that preceded it requires access to a wide swath of information not immediately available in any single location. As a result, individuals and groups working on after-action reviews or codifying lessons learned for system improvement are often working without the full evidence base that could optimize their work.

Access to the large yet fragmented body of global health security work is paramount to provide a historical picture of efforts to prevent and prepare for biological threats. Particularly in the context of COVID-19, it will be essential to undertake after-action review, address gaps in preparedness, and craft forward-looking strategies informed by past lessons. To support these efforts, we developed Health Security Net,^6^ a centralized repository of expert-curated global health security resources. Health Security Net contains more than 2,000 collated and annotated resources, which are publicly available online at healthsecuritynet.org.

## Methods

### Identification of Digital Databases and Document Curation

To assemble this database of research, oversight, guidelines, and plans, we first undertook an evaluation of preexisting digital databases relevant to the field. Based on expert recommendations, we identified a set of digital collections from which the research team gathered records. These collections included the World Health Organization Institutional Repository for Information Sharing (IRIS)^7^; the United Nations Digital Library^8^; United Nations Security Council meeting records^9^; the World Organisation for Animal Health Documentation Centre^10^; the US Government Accountability Office Action Tracker and Recommendations databases^11^; the Congressional Record^12^; the Congressional Research Service^13^; and the National Academies of Science, Engineering, and Medicine Research Center (under the Biology and Life Sciences and the Health and Medicine topics)^14^; among others. To extract relevant records from these databases, we developed a standardized set of search terms to identify documents relating to global and national awareness of emerging infectious disease or other naturally occurring pandemic threats and the risk from those threats. These 26 search terms (Box) encompassed key areas of interest within the field, including critical activities related to preparedness (eg, “biosurveillance,” “medical countermeasures,” “medical supply chain security”), commonly used terms (eg, “emerging infectious disease,” “health security,” “pandemic,” “biodefense”), and select pathogens or diseases with pandemic potential (eg, “Ebola,” “coronavirus,” “influenza,” “MERS,” “SARS”). As we reviewed the records, we excluded items focused only on endemic diseases or those typically addressed by the global health (as opposed to global health security) community, such as HIV, tuberculosis, malaria, and noncommunicable diseases, as well as fact sheets on diseases. Resources related to bioterrorism and biosafety or biological accidents were only included if, in expert judgment, the item made a substantial contribution to understanding the global health security field or a specific pandemic threat.

**Table:** Box. Keywords used for literature review

Biodefense
Biological threat
Biopreparedness
Biosurveillance
Biothreat
CBRN
Chemical, biological, radiological, and nuclear
Coronavirus
Ebola
Emerging infectious disease
Global infectious disease
Health security
Infectious disease epidemic
Influenza
MCM
Medical countermeasure
Medical preparedness
Medical readiness
Medical supply chain security
MERS
Middle east respiratory syndrome
Pandemic
Public health response
SARS
Severe acute respiratory syndrome
Zika

Limitations to this systematic approach included the sheer quantity of results produced and sites that restricted batch exports or lacked text matching. For instance, IRIS hosts more than 200,000 global health records dating from the 1800s and is the most specific source available for global health documents.^7^ Only a subset of these, however, helps tell the story of global health security and readiness for outbreaks with pandemic potential. Furthermore, these documents are distributed throughout the expansive database and not readily accessible via the given IRIS filters. Searches using the predefined terms yielded thousands of results, although IRIS only has the capability to export 500 documents at a time. Given time and resource constraints, researchers were required to identify relevant Medical Subject Headings (MeSH)^15^ subject categories in IRIS, and manually review only the documents within those identified categories. Many of the same search challenges with IRIS persisted across other organizations' websites, reflecting the difficulty stakeholders may face when trying to work across and within these libraries to assess preparedness. Similar limitations for other sites necessitated the use of a subset of the keyword search terms to consult gray literature, including within United Nations organization websites (ie, United Nations General Assembly documentation,^8^ United Nations Security Council meeting records,^9^ Food and Agriculture Organization of the United Nations conference documents,^16^ World Health Assembly^17^) and the World Organisation for Animal Health Documentation Centre.^10^ To systematize the search for third-party reports (eg, nongovernmental organization, academic, private sector), we consulted the top 10 think tanks from the University of Pennsylvania Think Tanks and Civil Societies Program Global Go To Think Tank Index reports.^18^ Additional reports were captured through other methods, such as through Google searches for simulations and exercises, and review of reference lists within captured US Government Accountability Office reports. This step led to the capture of documents from organizations such as the American Hospital Association,^19^ the Bill & Melinda Gates Foundation,^20^ the Johns Hopkins Center for Health Security,^21^ and the Transatlantic Biosecurity Network.^22^ We conducted a limited search of the peer-reviewed literature in Ovid MEDLINE,^23^ parameterized to capture papers about coronavirus risk. Each record gathered includes a hyperlink to the source and, where permissible by the publisher and/or licensing permissions, a PDF of the publication. Detailed methods are available on the Health Security Net documentation page.^24^

### Development of Data Taxonomy

To ensure the documents gathered through the literature review are easily accessible and fully searchable for stakeholders, the research team developed a custom data taxonomy and data dictionary to define key metadata and organize the dataset. A total of 20 metadata elements were defined for each document, including information about each document's publication, funding, geographic scope, and applicability to global health security. A coder manually characterized each included document based on a detailed review of its content, including an original written description and hand-selected topic areas and tags. A second coder reviewed each document for internal consistency. If coders disagreed about the categorization of a document, a third researcher adjudicated the decision.

To aid end users in identifying broad areas of interest across the preparedness spectrum, topic areas were chosen that stem from the core elements of health emergency preparedness to prevent, detect, and respond to infectious disease threats (eg, strategic planning, threat/risk awareness, disease surveillance/detection, medical preparedness/emergency response, international aid/collaboration). Each record was categorized into a single topic area reflecting the predominant topic of that record; if multiple topic areas were applicable, the coder was instructed to choose the most relevant, and the decision was confirmed by a second coder. When no topic areas applied or the record was broad, coders chose the “Other” category. Combinations of topic areas were not supported in the interest of straightforward resource categorization, visualization, and exploration. However, the inclusion of tags and a search bar in the online tool help address search limitations imposed by the single-select topic area. Tags, or subtopics, were defined based on recurring themes identified in the literature review and support sort and filter of all documents for end users to refine the results. Multiple tags were applied to single resources to best characterize its contents. Finally, a search bar was added to Health Security Net to give users full flexibility in their search. Users can enter any term into the text field to search for that keyword in resource titles, descriptions, and the first 1,000 characters of an attached PDF if one is available. Topic areas, tags, and the search bar all exist independently of one another; researchers using Health Security Net could use any combination of these features to filter based on the level of specificity of their search. The full taxonomy is described in detail in the site's documentation and glossary of terms.

Finally, we developed an ontology to link public health events to documents with content about that event. We included all public health emergencies of international concern, excluding polio, and events not declared or predating the public health emergencies of international concern declaration process that we assessed to meet the definition of an event per International Health Regulations Annex 2. Documents were categorized by event based on relevance to the topic independent of date of publication. This structure enables researchers to review the activities taken during different stages of a public health emergency in the context of lessons learned and after-action reviews.

## Analysis

With a robust and growing selection of hand-curated and coded documents, Health Security Net unites the once fragmented body of global health security literature as it relates to pandemics and other high-consequence infectious diseases in a way that can support the international community's efforts to understand and track who knew what, when, and which actions were taken with regard to health emergency preparedness. Health Security Net now contains more than 2,200 documents published from 1972 to the present, including assessments, oversight efforts, white papers, guidelines, strategies, and implementation plans. The contents of these documents primarily focus on pandemic-scale threats, but also encompass resources that address the global threat of emerging infectious disease, biological threat and risk awareness, global public health vulnerabilities, and outbreak-specific responses for 10 infectious disease events.

To guide users interested in particular topics, the researchers categorized each record by broad topic area, such as risk awareness or disease surveillance. While many records cover a variety of topics, the majority of resources lend a focus to medical preparedness and response (n = 945, 42%) compared with other topics like threat/risk awareness (n = 218, 10%) or disease surveillance/detection (n = 283, 13%). Nearly a quarter of the database's records contain the word “flu” or “influenza” in the title, suggesting a historical emphasis on influenza pandemic awareness and planning. Among records that relate to specific outbreaks before COVID-19, Ebola accounted for the most reports of any outbreak studied. For its part, the US Congress met 167 times over 25 years in committee hearings on pandemic-related matters, often in response to ongoing outbreaks. Deeper scholarly study of these and related records could help reveal what drove these trends, how perceived risks drove decisionmaking, and what impact these decisions have made on pandemic readiness over time.

## Discussion

This living repository of documents is designed to be an initial curation of the body of literature in health security for researchers and practitioners to identify and access the core documents at the foundation of the field. Additional and more in-depth analysis of the contents may reveal whether efforts across threat awareness, prevention, detection, response, and recovery appropriately addressed the risk landscape. Interdisciplinary analysis may be especially revealing for understanding dynamics of funding, policy, politics, and governance, and the ways in which they interact in the context of preparedness. The library is integrated within the Georgetown International Disease and Events Analysis platform, a suite of tools that include datasets on financing health security, preparedness scores, historical records of outbreaks, and outbreak activities that will help inform such comprehensive, cross-disciplinary evaluations.^25^

Because the library contains only what was accessible to its developers, Health Security Net is subject to selection biases. We have mitigated individual biases by employing interdisciplinary researchers with expertise in different regions and aspects of health security, consulting with colleagues from within the field for feedback, and encouraging users to submit their own additional documents for inclusion. Notably, however, there remains an immensity of work at the global scale and in non-English languages that have yet to be explored by our research team.

Secondly, as global health security continues to gain traction in the wake of the COVID-19 pandemic, we expect that it will be increasingly difficult to keep up with documents before old plans, policies, or evaluations are replaced with new ones. Thus, there are inevitably important documents our team will not capture, limiting our ability to construct a fully exhaustive narrative of preparedness and response efforts. In an effort to keep pace with knowledge production, our team captures new documents on a weekly basis and uses Google alerts with our curated search terms to monitor new content from sources we may not have reviewed yet. The research team also meets to identify and prioritize high-impact events, such as travel bans as a result of the Omicron variant, to inform targeted expansions of the library to ensure that Health Security Net covers the most important resources and research.

Another major challenge with any digital library is ensuring that documents already captured in the database have working hyperlinks. On a quarterly basis and when alerted to an issue, our team checks the URLs of all captured resources. As the collection grows, however, it may become more challenging to provide upkeep for broken links. As a safety measure, we use permanent links where possible. For items whose URLs have been deprecated and a DOI is not available, the PDF is stored using the application programming interface in a secure database so that the information is not lost.

Lastly, data collection was influenced by funder priorities in learning about the development of preparedness efforts leading up to COVID-19. At the time of writing, COVID-19 is an ongoing emergency and thus, academic literature and search platforms are oversaturated with information about the outbreak. This holds true for many public health emergencies; it is well-documented that governments, organizations, and researchers renew their commitments to health security during and right after an outbreak. Eventually, however, this funding tapers off as the pandemic threat is forgotten, causing a skew in the literature toward response efforts over preparedness efforts. It is only through uniting this body of work that these gaps can be readily identified and improved upon. Our research team is actively focused on expanding Health Security Net to include new documents on risk assessments, strategic planning, and capacity strengthening that could inform the targets of preparedness funding. We also encourage any researchers or organizations with resources or studies that they feel would be a meaningful addition to Health Security Net to contact the research team.

## Future Research Directions

We plan to undertake another literature review, prioritizing countries that mounted substantial or unique COVID-19 responses, given the field's current research priorities. Using each country's peripandemic activities, we intend to capture the most important health security documents from the prioritized countries in the country's official language, and in English where possible. In the longer term, this effort will expand to other countries and outbreak events.

The policy, public health, and academic communities have much to learn from what was and was not anticipated or articulated in the years preceding COVID-19. Doing so may help us understand the key question of whether we could have been better prepared for the challenge that COVID-19 presented. To contextualize COVID-19 within the frame of historical efforts—as revealed by thousands of publications now housed in this historical database—could provide perspective on why the pandemic unfolded as it did, while helping the global community better prevent and prepare for the next biological threat.
